# Varying Manpower Alters Dental Health in a Developing Health Care System

**DOI:** 10.1016/j.identj.2021.04.003

**Published:** 2021-06-13

**Authors:** Fariborz Bayat, Miira M. Vehkalahti, Alireza Akbarzadeh, Farshid Monajemi

**Affiliations:** a Dental Research Center, Research Institute of Dental Sciences, Shahid Beheshti University of Medical Sciences, Tehran, Iran; b Department of Oral and Maxillofacial Diseases, University of Helsinki, Helsinki, Finland; c Proteomics Research Center, Department of Biostatistics, School of Allied Medical Sciences, Shahid Beheshti University of Medical Sciences, Tehran, Iran

**Keywords:** Developing health care system, Dentists-population ratio, DMFT index, Oral health care workers, Prevention, Workforce

## Abstract

**Objectives:**

This study assessed relationships between oral health care workforce and dental health in 12-year-olds in a developing health care system in Iran from 1992 to 2014 and compared these findings with the most recent corresponding findings in selected countries.

**Methods:**

Data regarding oral health care workers from 1962 to 2014 were extracted from the comprehensive human resource data bank of the Shahid Beheshti Research Institute of Dental Sciences. Data regarding decayed, missing, and filled permanent teeth (DMFT) of 12-year-olds, extracted from official statistics, described dental health. Comparisons with other countries utilised the database of the World Health Organization. Changes in the DMFT index with fluctuations in the number of oral health care workers were investigated using exploratory data analysis methods. Associations of DMFT with the density of the oral health care workforce were evaluated using a multiple linear regression model.

**Results:**

The trend in supply of dental workforce in Iran began to expand in the 1970s and, after a reduction in 2003 to 2007, reached a peak by 2014. Means of DMFT indices of 12-year-olds in Iran fluctuated between 1.50 and 2.40 from 1992 to 2014. The relationship between the dentist to population ratio and the DMFT index of 12-year-olds showed a downwards trend (*r* = −0.994; *P* < .001) until 1998 and afterwards an upwards trend (*r* = 0.887; *P* < .001). Globally, the DMFT index decreased in countries with a preventively-oriented oral health care workforce.

**Conclusions:**

Increased numbers of dentists have no significant impact on improving dental health in 12-year-olds. To promote dental health, the system providing health services should implement a preventively-oriented approach when planning for the oral health workforce.

## Introduction

Adequate public access to health services is one strategy to decrease the burden of disease and improve quality of life.^1^^,^^2^ One important factor to achieve optimal health is a sufficient number of knowledgeable and trained staff in a health care system. The number and type of oral health care workers (OHWs) and the method of service provision by them at different levels are highly debated topics by health care authorities, policy makers, and educational organisations worldwide.^1^^,^^3^^,^^4^

Composition of the oral health workforce between developed and developing countries shows marked differences, particularly in the numbers of dentists, specialists, dental auxiliaries, dental schools, and graduates per year. Development of oral health services to match the needs of the population, training programmes for personnel to match the oral health needs and the infrastructure of the country, and ensuring an even distribution of this workforce throughout the country have, for many years, been of great concern.^4^ The importance of these issues has become evident in many countries where the education of dentists appears inappropriate to the oral health needs and demands.^3^

An increased number of dentists may not serve as a solution for unmet oral health needs if they are distributed unevenly.^5^ Having an adequate number of OHWs to serve the needs and demands of the population is an important indicator of the effectiveness of a health care system.^6^ The importance of preventive measures is known to maintain dental health at the person level, but less is known at the population level about preventive strategies and their impact on dental health.^7^ The World Health Organization (WHO) first implemented worldwide use of the index of decayed, missing, and filled permanent teeth (DMFT) of 12-year-olds and later proposed dental health indicators to be used for adults in index age groups of 35- to 44- and 65- to 74-year-olds. In Iran by use of the DMFT index, oral health status has been evaluated in several reports.^7^, ^8^, ^9^, ^10^, ^11^, ^12^, ^13^

This study assessed the relationships between the oral health care workforce and dental health in 12-year-olds in a developing health care system in Iran from 1992 to 2014 and compared these findings with the most recent corresponding findings in selected countries.

## Methods

### Data collection

In this health service research study, data regarding OHWs were extracted from the comprehensive human resource database of the Shahid Beheshti Dental Research Center.

The database was established and maintained according to a contract between the Oral Health Office of the Ministry of Health (MOH) and the Research Institute of Dental Sciences^14^ which prepared software for oral health managers to be able to access the admission, graduation, and employment of oral health personnel and to evaluate and plan the utilisation of OHWs. Resources for collecting the data, updated every year, were primarily offered by the MOH, medical universities in the provinces, the Medical Council of Iran, the Association of Dental Nurses and Hygienists, and the Iranian Dental Technologist Association.

The data included numbers of various types of oral health care workers by sex in 2014. In addition, the numbers of general dentists, specialists, and dental auxiliaries were collected to illustrate trends in supply of dental workforce in Iran from 1962 to 2014.

To describe dental health, the indices of DMFT and of decayed permanent teeth (DT) of 12-year-olds were extracted from relevant published studies^8^^,^^9^ and the national reports by the Oral Health Department of the MOH in Iran.^10^, ^11^, ^12^, ^13^

According to the dentist to population ratio, 9 countries were selected using the WHO Oral Health Country/Area database profile to extract the related data.^15^ The main criterion for selecting countries was accessibility to the following information: DMFT in 12-year-olds and detailed information of dental workforce (dentists and dental auxiliaries). Based on the manpower information, 3 categories were defined and 3 countries selected of each:

1 Countries with a high dentist to population ratio of around 1:1000 such as Japan, Finland, and Denmark.
2 Countries with a medium dentist to population ratio of around 1:1500 to 1:2000 such as Australia, the United States, and the UK.
3 Countries with a low dentist to population ratio starting around 1:3000, such as Turkey, Iran, and Saudi Arabia.

### Statistical methods

The pattern of change in DMFT index with fluctuations in dental workforce was investigated using exploratory data analysis methods. Correlation coefficient was estimated for associations between DMFT indices of 12-year-olds and the number of dentists between 1992 and 2014. Further, the relations between DMFT (dependent variable) and number of dentists per 100,000 population, number of preventive staff per 100,000 population, and the dentist to preventive staff ratio were evaluated from1998 to 2014 using a multiple linear regression model.

## Results

Table 1 shows the number of OHWs in Iran according to their academic education and sex by the end of 2014. Accordingly, by the end of 2014, the total number of general dentists registered in the Medical Council of Iran was 23,263; of these, 58% were male and 42% female. This value translated to 36 dentists per 100,000 population.

**Table 1:** Structure of oral health workforce by their academic education and sex in Iran in 2014.

Academic education	All		Women	Men
	n	%	%	%
Dentists (GPs)	23,263	84	42	58
Specialists	3722	13	49	51
Postgraduate students	755	3	65	35
Dental auxiliaries				
Dental therapists	39	3	10	90
Dental hygienists	1200	75	85	15
Oral hygienists	352	22	48	52

**Fig. 1:**
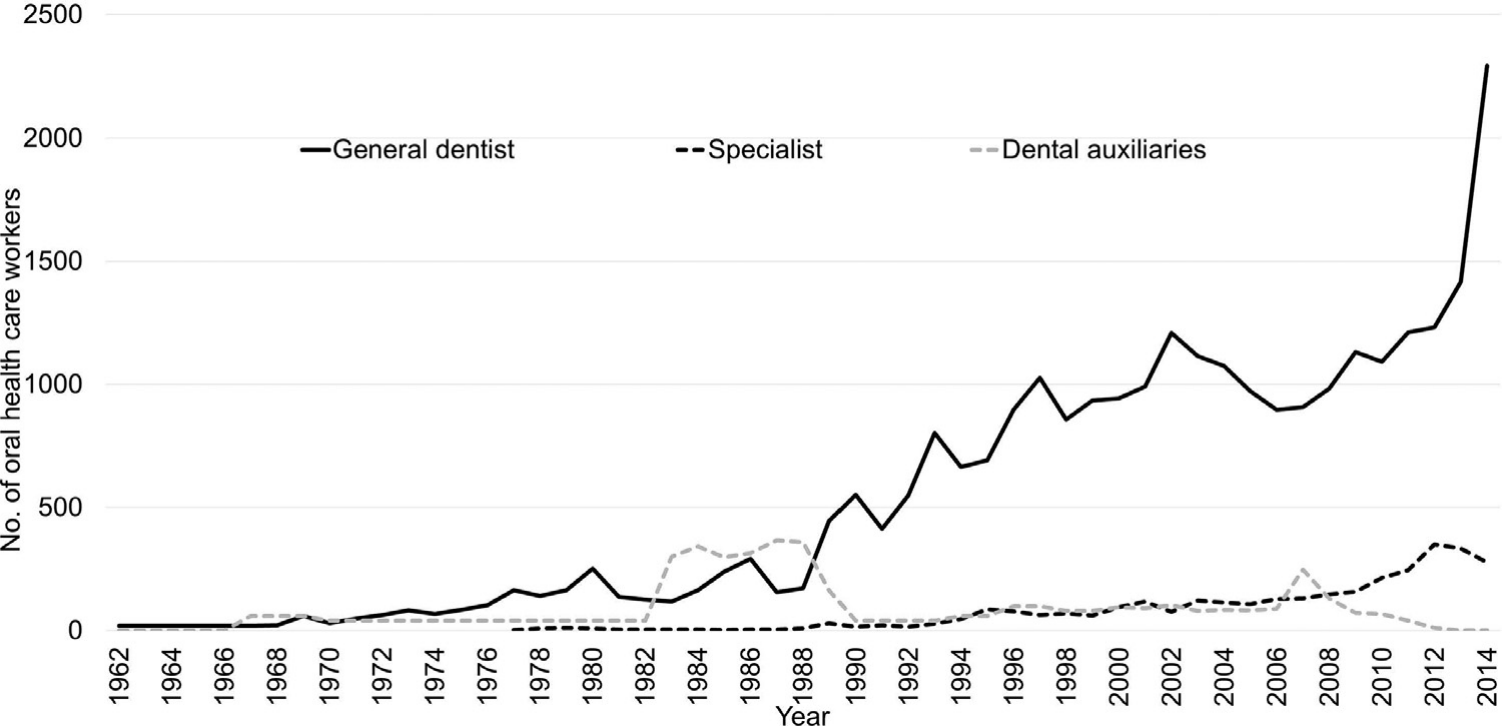
Trend in the yearly supply of dental manpower in Iran in 1967–2014.

Figure 1 shows the trend in supply of dental workforce in Iran during 1962 to 2014. The establishment of dental schools and supply of general dentists had a steady pattern until 2002 (18 dental colleges nationwide). A reduction in supply was noted from 2002 to 2007, but over the next 7 years (2008–2014) there was a change in the number of graduates. Since 1980, the supply of dentists can be divided into two distinct periods. From 1980 to 2002, the number of graduates was almost constant, but from 2002 to 2014 the number of graduates increased from 1210 to 2294 per year due to the establishment of new dental schools (an increase from 18 to 48 dental colleges).

The trend of admission of dental auxiliaries was the opposite of that of dental students and postgraduate dental students. The number of dental auxiliaries was fewer than 1000 by 2014 with the following trend: dental clinicians: 39, oral hygienists: 750, and dental hygienists: 137.

**Fig. 2:**
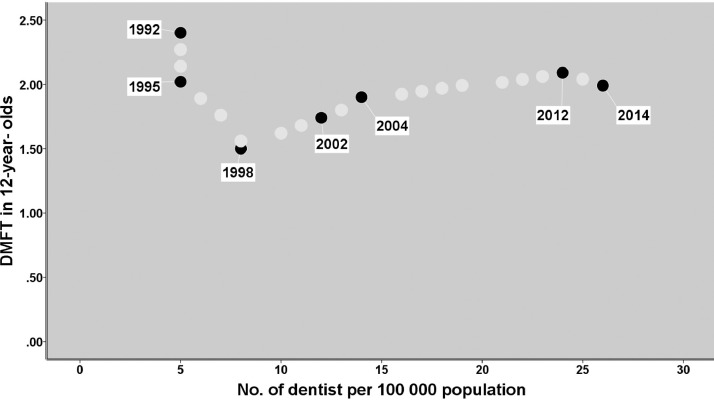
Relationship between DMFT in 12-year-olds and number of dentists 5 years earlier per 100,000 population in Iran in 1992–2014. The black circles are for the official DMFT values, the light-grey circles for the statistically estimated DMFT values.

Means of DMFT indices of 12-year-olds in Iran fluctuated between 1.50 and 2.40 from 1992 to 2014 (Table 2). Figure 2 presents the relationship between the DMFT index of 12-year-olds and the number of dentists per 100,000 inhabitants during the years 1992 to 2014. The results showed a significant inverse correlation until 1998 (*r* = −0.994; *P* < .001) and a significant positive correlation (*r* = 0.887; *P* < .001) from 1998 to 2014 between the dentist to population ratio and the DMFT index of 12-year-olds.

**Table 2:** The index of decayed, missing, and filled permanent teeth (DMFT) of 12-year-olds in Iran from 1992 to 2014.

Year	DMFT	Author (year)
1992	2.40	Jaber Ansari Z (1998)
1995	2.02	Samadzadeh H, Hessari H, Nouri M (2001)
1998	1.50	MOH, Oral Health Department (2000)
2002	1.74	MOH, Oral Health Department (2004)
2004	1.90	MOH, Oral Health Department (2004)
2012	2.09	MOH, Oral Health Department (2012)
2014	1.98	MOH, Oral Health Department (2014)

MOH, Ministry of Health and Medical Education, Iran.

A multiple regression model analysing relationships from 1998 to 2014 between the DMFT index of 12-year-olds and workforce characteristics (Table 3) shows that the dentist to preventive staff ratio has a negative effect on DMFT index, with a standardised coefficient of −2.2 (*P* = .017). This means that a higher dentist to preventive staff ratio results in a lower DMFT index, while a higher number of dentists per 100,000 population has a positive effect on DMFT, with a standardised coefficient of 3.1 (*P* = .001).

**Table 3:** Relationship between the index of decayed, missing, and filled permanent teeth (DMFT) of 12-year-olds and manpower characteristics in Iran 1998–2014.

Manpower characteristics	β (SE)	Standardised coefficient	*t*	*P*
Constant	0.634 (0.35)		1.80	.096
Dentist to population ratio	0.096 (0.02)	3.087	4.40	.001
Preventive staff to population ratio	0.023 (0.07)	0.048	0.30	.764
Dentist to preventive staff ratio	−0.242 (0.88)	−2.171	2.75	.017
R square = 0.937 Adjusted R square = 0.922				

As observed in Table 4, Japan shows the lowest DMFT index (0.2) and Saudi Arabia the highest (2.8). However, the dentist to population ratio in Denmark is the highest (1:1086) and in Saudi Arabia the lowest (1:7856), followed by Turkey (1:3420) and Iran (1:2908).

**Table 4:** Comparison of dental manpower and the indices of decayed, missing, and filled permanent teeth (DMFT) and decayed permanent teeth (DT) of 12-year-olds in selected countries.

Characteristics of countries	Japan	Denmark	Finland	Australia	USA	UK	Turkey	Iran	Saudi Arabia
Population (millions)	127	5.6	5.5	24	324	64	81	81	32
DMFT/DT in 12-year-olds	0.2/0.0	0.4/0.1	0.7/0.3	1.0/0.4	0.12/NA	1.3/0.4	1.9/1.7	2.0/1.6	2.8/2.6
No. of active dentists	99 686	5161	4500	15 764	196 441	34 534	22 996	26 985	4073
Female dentists (%)	18	58	68	39	30	56	41	43	NA
Dentist to population ratio	1:1274	1:1086	1:1208	1:1524	1:1654	1:1850	1:3420	1:2908	1:7856
No. of specialists	NA	388	672	1841	39 207	4179	NA	4453	NA
No. of dental auxiliaries^a^	73 297	2000	1490	3988	200 532	8631	0	789	0
Dental auxiliary to dentist ratio	1:1.4	1:4	1:4	1:4	1:1	1:4.5	NA	1:33	NA
No. of dental schools	29	2	4	12	66	16	28	48	28
Public	NA	2	4	12	38	16	23	34	18
Private	NA	0	0	NA	28	0	5	14	10
No. of dental graduates/year	2700	120	100	600	5811	1052	1706	2050	393

a Dental auxiliaries = dental hygienists and therapists Sources: Oral Health Database, Country Oral Health Profiles, available from: https://www.mah.se/CAPP/Country-Oral-Health-Profiles. Kravitz A, Bullock A, Cowpe J, Barnes E. (2014). Council of European Dentists manual of dental practice 2014 (Edition 5). Available from: https://www.researchgate.net/publication/263476582_Council_of_European_Dentists_MANUAL_OF_DENTAL_PRACTICE_2014_Edition_5. Current World Population, available at: http://www.worldometers.info/world-population.

The dental auxiliary to dentist ratio varies between the selected countries; the US (1:1) and Japan (1:1.4) have the highest ratios, whereas there are no dental auxiliaries in Turkey and the number is unavailable in Saudi Arabia.

## Discussion

This assessment regarding the dental health status of the 12-year-old Iranian population from 1992 to 2014 was based on official statistics demonstating that the DMFT index first decreased but then returned almost to its initial level. Data on the oral health workforce revealed a significant increase in numbers of dental graduates in the past decade, while the numbers of dental auxiliaries decreased. An increase in the number of dental clinicians alone did not have a significant positive impact on improvement on the oral health of the public. Evaluation of the impact of various factors on oral health in selected countries highlighted the importance of a prevention-oriented oral health workforce.

Countries with a well-established health care system generally have a preventive programme for their population,^16^ such as Japan,^15^ and reduce the cost of care, such as in Finland and Denmark.^15^^,^^16^ In Australia,^15^^,^^18^^,^^19^ a significant improvement in oral health status of the public is attributed to implementation of preventive programmes at the national level, propagating the use of fluoridated toothpastes and management of a service provision system such as use of school dental services. Dental hygienists play an important role in implementation of these programmes.^20^ In the UK,^15^^,^^17^ the care centres often have a combination of human resources at different levels, providing services based on their expertise. Preventive measures are likewise implemented at the national level. In the US,^15^^,^^21^^,^^22^ around 69% of the population uses fluoridated water, and some specific programmes are implemented at the national and state levels. Dental hygienists can enhance the provision of preventive services in dental offices and clinics.^23^^,^^24^

The absence of health-based programmes at the national level and low number (Iran) or absence (Turkey and Saudi Arabia) of intermediate providers in the oral health care system (despite the high number of dentists) are associated with a higher DMFT index (Table 4). In Saudi Arabia, despite the provision of primary health care services and centres providing private and insurance-covered dental services,^25^ no specific programme has been implemented at the school level to promote oral health. The level of usage of oral health measures is low, especially in children and adolescents. People are reluctant to care for their teeth and are not interested in preventive measures due to their low level of knowledge about oral health.^25^, ^26^, ^27^, ^28^, ^29^, ^30^ Further, the main factors causing a reduction in rate of caries, such as fluoridation of drinking water and tooth brushing, are virtually nonexistent in people's lifestyles,^31^^,^^32^ whereas these interventions, when applied, greatly contribute to promotion of dental health.^33^^,^^34^

In Turkey,^15^ intermediate human resources are lacking and there is no national programme for oral health promotion. However, the national health insurance system started to include dental care measures in 2008, aiming to encourage citizens to receive preventive care. Nevertheless, the services provided to 5- to 15-year-olds are mainly therapeutic rather than preventive.^35^ The current preventive programme in Turkey is the “tooth protection days” camping programme for 6- to 12-year-olds, implemented by the Turkish Dental Association.^35^ Since 2008, a school-based health promotion programme has been employed in some provinces of Turkey.^36^

Policies and programmes of the health care system in Iran with respect to changes in oral health indices and number of dentists in the past 20 years have occurred over 3 time periods (1992–1998, 1998–2012, 2012–2014). In the first period (1992–1998), the required personnel were recruited in line with implementation of oral health promotion policies. At the same time, in order to supply OHWs, dental hygienists and general dentists were recruited as part of their mandatory postgraduate service. In 1995, oral health was included in the primary health care system, and national programmes were implemented at the elementary school level, with the cooperation of the government.

Reasons for increased DMFT in the second period (1998–2012) may include (a) downgrading the position of the Oral Health Office of the MOH in policy making and supervision of oral health programmes early in this period, (b) onset of gradual exclusion of dental hygienists from the network and discontinuing the admission of students to these programs, (c) reduction followed by cessation of admission of dental auxiliaries, and (d) increase in the number of dental schools due to political pressure requiring establishment of at least one dental school in each province. That again expanded the number of dental students and led to dental schools’ self-regulation and autonomous management.

Reduction in DMFT at the onset of the third period (2012–2014) can be due to the change in the national health care system of Iran and reimplementation of oral health promotion programmes in line with the national health promotion plan, which was associated with activity of OHWs in schools. On the other hand, a most striking increase in numbers of dentists was found between 2012 and 2014, probably due to numerous overseas graduates accepted by the MOH to the dental profession. After 2014, the approval required passing the comprehensive national dental examination. Furthermore, the quality of the new dental schools was evaluated by the Specialised Council for Dental Education.

Provision of preventive measures is included in the dental curricula, and dental students^37^ and dental practitioners^38^^,^^39^ in Iran have adequate knowledge. Nevertheless, they often are reluctant to provide preventive services and oral hygiene instruction to patients, maybe due to the great emphasis placed on therapeutic services in dental curricula while preventive services are considered less important services. Moreover, the strong attitude of dentists regarding preventive care reinforces the knowledge and serves as the foundation for the service provided.^40^ National dental curriculum revisions in Iran were conducted in 1982, 1988, 1999, and 2018. In the recent revision, an emphasis on preventive dentistry has been added.^41^^,^^42^

Dental clinicians mostly believe that preventive care is not cost-effective^43^^,^^44^ and perceive it as not very rewarding.^38^^,^^45^ The insurance coverage for preventive care by the government or insurance companies also affects dentists’ provision (or lack thereof) of preventive care.^46^, ^47^, ^48^ It is noteworthy that the primary goal of dental education is to acquire technical and biological skills for treatment of patients.^49^ A high prevalence of caries in patients younger than 18 years, despite increased numbers of dentists, indicates that this approach does not have a significant effect on promotion of public oral health (nor does the restoration of carious teeth in many developed countries), and the main focus should be shifted to prevention.^50^

Strengths and limitations exist regarding this study. The nationwide data of OHWs that was used can be considered high-grade and reliable since it is register-based. The DMFT data for 12-year-olds extracted from the WHO statistics was based on nationwide studies or on local studies over a variable number of years. This may adversely effect the validity and reliability of data and thus represents a limitation of the study.

## Conclusions

Increased numbers of dentists have no significant impact on improving dental health in 12-year-olds. To promote dental health, the system providing health services should implement a preventive-oriented approach when planning for the number of oral health providers.

## Conflict of interest

None disclosed.
